# An unusual presentation of extragenital lichen sclerosus—An extensive keratotic variant

**DOI:** 10.1002/ski2.341

**Published:** 2024-03-21

**Authors:** Mario Magaña, Carol Estefania Márquez‐Maldonado

**Affiliations:** ^1^ Faculty of Medicine UNAM Universidad Nacional Autonoma de Mexico Mexico City Mexico; ^2^ Service of Dermatology Hospital General de Mexico “Dr. Eduardo Liceaga”, S. S. (Ministry of Health) Mexico City Mexico; ^3^ Service of Dermatology Centro Médico Nacional 20 de Noviembre, ISSSTE Mexico City Mexico

## Abstract

In this case report, we outline a case of a 71‐year‐old woman who presented to the dermatology clinic with a history of keratotic and atrophic warty‐like plaques on her lower limbs, which limited the movements in her right lower extremity. A skin radial biopsy of one of the plaques was performed and the diagnosis of extragenital lichen sclerosus (ELS) was established. She underwent an anogenital exam and fortunately, lesions were not found. The patient in this case showed an unusual variant of ELS.

## Graphical abstract

We outline a case of a 71‐year‐old woman who presented a keratotic and atrophic warty‐like plaques on her lower limbs, which limited the movements in her right lower extremity. A skin radial biopsy of one the plaques was performed and the diagnosis of extragenital lichen sclerosus was established. The patient in this case showed an unusual variant with exuberant clinical features in the lower limbs.

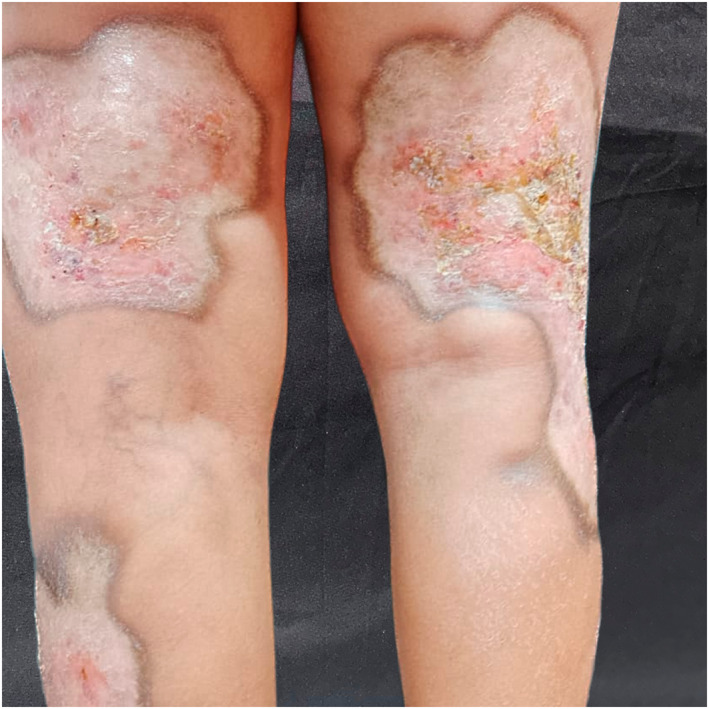

## 1 INTRODUCTION

Lichen sclerosus (LS) is a chronic, inflammatory, and progressive disease.^1^ The aetiology is unknown and the pathogenesis is unclear.^1^ The prevalence rate of LS varies from 0.1% to 1.67%.^1^ It has a high predilection for the anogenital area about 85% of total cases, while extragenital presentations represent <15%.^2^ It preferentially affects women after menopause.^2^ Clinically it appears as hypopigmented plaque in the trunk.^1^ It is also associated with a higher risk of vulvar malignancy.^3^ We present an atypical presentation case of this disorder of a 71‐year‐old woman who developed extensive extragenital lichen sclerosus (ELS) in her lower limbs; to contribute to the identification of clinical variants.

## 2 CASE REPORT

**FIGURE 1:**
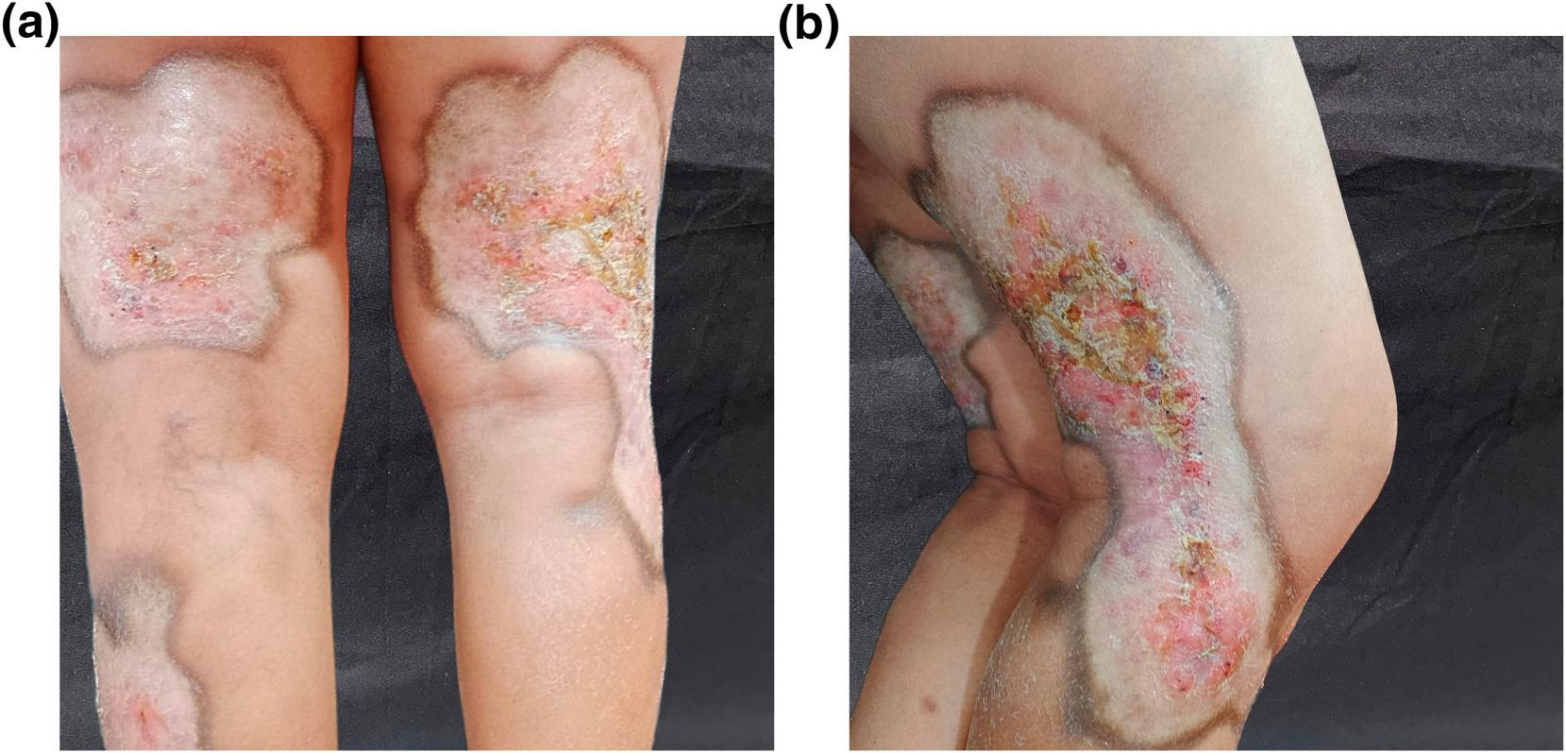
(a) Ivory‐white warty‐like plaques on the lower limbs. (b) The lateral view shows an extensive lesion in the lower limbs.

**FIGURE 2:**
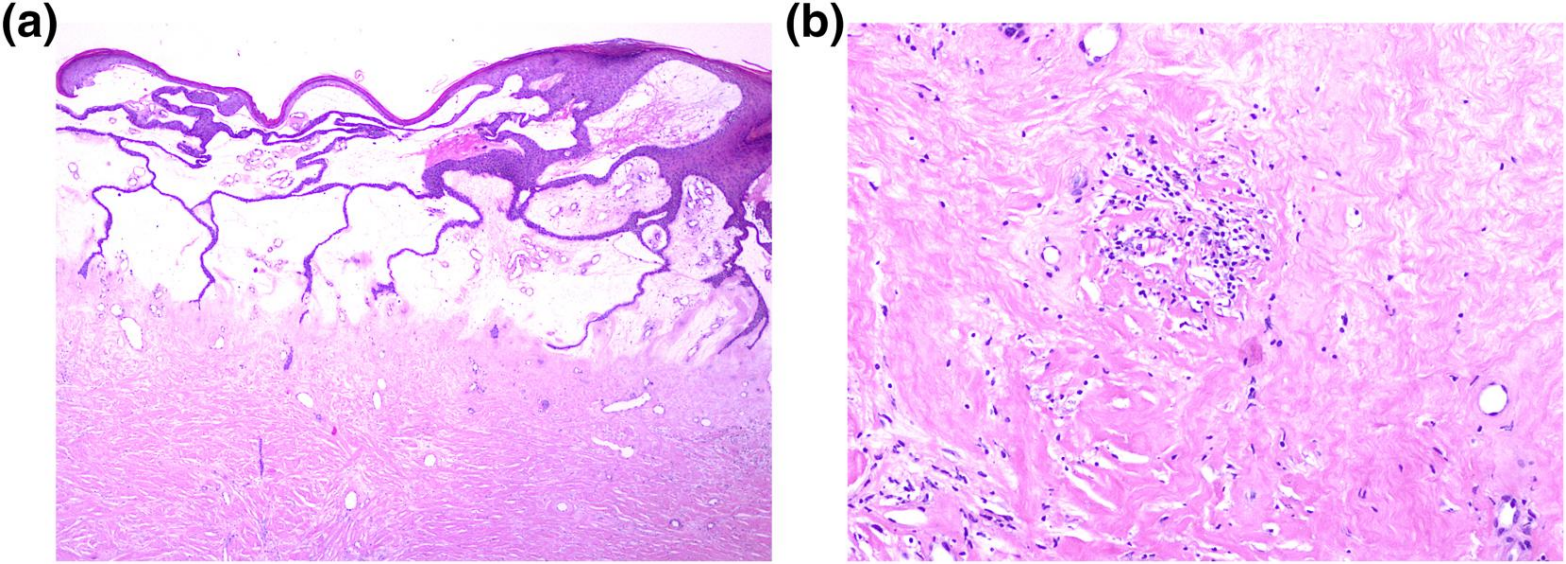
(a) Haematoxylin and eosin stain (40× magnification). Orthokeratosis, thinning of the epidermis, effacement of rete ridges, vacuolar alteration of the basal layer, and intense oedema in the superficial dermis with homogenisation of collagen and poor staining in haematoxylin and eosin. (b) Collagen fibre hyperplasia, hyalinisation, and sclerosis of the whole dermis. There is dilatation of thin‐walled vessels in the zone and beneath the oedema there is a diffuse interstitial and perivascular lymphoplasmacytic infiltrate in the mid‐dermis (100× magnification).

**FIGURE 3:**
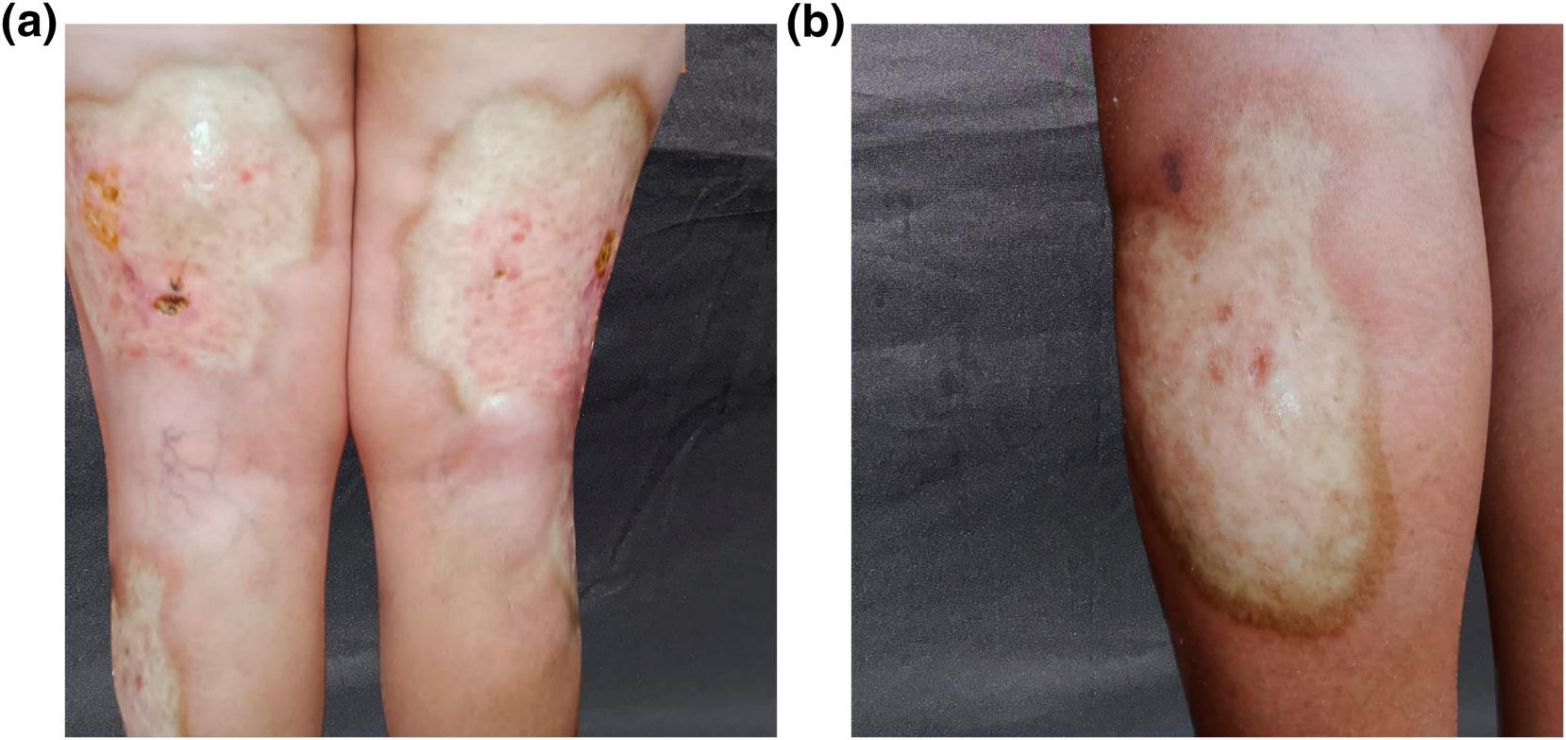
(a, b) Significant improvement with treatment after 5 months of treatment.

A 71‐year‐old woman, without past medical history, was consulted for multiple keratotic and atrophic plaques in her lower limbs, with movement limitation in her right lower extremity and occasional pain of a 2‐year evolution. Meanwhile, the skin had gradually thickened and hardened on the lower limbs, with mild itching. Physical examination revealed a symmetrical and bilateral process, involving the lower limbs, compounded by multiple ovals, hypopigmented and keratotic plaques with a warty appearance in its centre and atrophy in the periphery; predominantly on the dorsum of thighs and legs; with hyperpigmented and defined edges, measuring 10 cm by 25 cm (Figure 1). The genital region was unaffected. Laboratory tests were ordered, including tuberculin skin test; all were unremarkable. A skin biopsy was performed and, reported orthokeratosis, epidermal atrophy, vacuolar alteration of the basal layer, and hyalinisation of the papillary dermal collagen with interstitial and perivascular lymphoplasmacytic infiltrate below (Figure 2). The diagnosis of ELS was concluded. She was treated with topical ointment clobetasol (0.05%) and tacrolimus (0.1%) twice a day on alternating days and oral prednisone 25 mg twice a day. She showed partial regression of the keratosis since the first month of treatment. Afterwards, systemic glucocorticoid tapering was started in the second month until the achievement of 25 mg twice a week with significant improvement (Figure 3). A multidisciplinary approach was applied joined with physiotherapy to improve her right extremity mobilisation.

## 3 DISCUSSION

LS is a chronic, inflammatory disease mostly observed in women.^4^ It was first coined by Henri Hallopeau in 1887.^1^ It has been known by different designations, but most of the literature case reports name it as ‘lichen sclerosus et atrophicus’.^1^ However, in 1976 the International Society for the Study of Vulvar Disease officially replaced it as LS.^1^

The LS prevalence is uncertain and underestimated.^2^ Extragenital‐isolated LS involvement is extremely rare, unlike genital LS.^2^ It could be associated with oral or genital LS and morphea, therefore a complete physical examination is imperative.^5^, ^6^ The pathogenesis has not been elucidated, however, proposed etiologic hypotheses include immune dysfunction, genetic predisposition, infectious agents, and trauma (Koebner phenomenon).^7^

ELS could affect any body site, but the trunk and lower limbs are the most commonly affected extra‐gential areas.^2^ Generally, it is characterised by atrophic, ivory‐white plaques, however, not all cases exhibit atrophy in histological findings.^2^ The histopathology shows an established lesion where this zone becomes more sclerotic in appearance and basement membrane thickening can also occur in later lesions.^8^

Several clinical presentations are depending upon distribution (generalised, palmoplantar, oral, blaschkoid, and linear) and morphology (bullous, ulcerative, annular, corymbiform, desquamative, telangiectatic, verrucous/keratotic and vitiligoid).^1^ The keratotic variant is considered an atypical variant.^9^ There have been few cases reported.^9^ Hence, the differential diagnosis is complicated by various overlapping clinical features of different infective and non‐infective conditions.

In this case, in the differential of ELS the keratotic morphology should be considered. Cutaneous T‐cell lymphoma, where lesions of patch‐stage mycosis fungoides may present with epidermal atrophy, but commonly associated with erythema, resembling disseminated eczema plaques.^4^ Morphea may resemble the classic, atrophic, white patches of ELS; and both can exist in the same lesion.^6^ Atrophic lichen planus lesions present violaceous, polygonal papules that converge in plaques with scale, mostly seen on the wrists or ankles, and frequently have intense itching.^1^, ^10^ Cutaneous deep mycoses such as chromoblastomycosis can show warty and hyperkeratotic‐like plaques and usually involve feet, knees, and lower legs.^11^ Sample direct examination with potassium hydroxide allows for evidence of fungal structures.^11^ Lupus vulgaris is a type of cutaneous tuberculosis that can mimic a variety of pathologies and can present keratotic warty‐like plaques, localised most on the face.^12^ Methods of detecting the mycobacteria as mycobacterial culture, stained smears, and biopsies, can support the diagnosis.^12^

The clinical course of the disease is variable and tends to be chronic.^4^ Currently, high‐potency topical glucocorticoids and calcineurin inhibitors are considered the mainstays of treatment.^2^, ^13^, ^14^ Other co‐adjuvants therapies are in study.^2^, ^14^ Otherwise, systemic therapy is primarily reserved for severe, extensive, refractory, or progressively worsening extragenital disease.^14^, ^15^ As well as in this case, each treatment should be individualised, emphasising the follow‐up in tailoring therapy trying to prevent progression and diagnose pre‐cancers at early stages.

## 4 PATIENT PERSPECTIVE

I was attended by several physicians and nobody could find a diagnosis. Unfortunately, I lost the previous diagnostic tests. The lesions before coming to the General Hospital ‘Dr. Eduardo Liceaga’ were progressing until became bigger and more painful. It was hindering my movements, sometimes I felt like a disabled person. After 5 months of treatment, I saw the difference, lesions were disappearing. Also, I had integral attention that included physiotherapy to improve my mobilisation.

## 5 CONCLUSION

We consider this report highlights the potential of ELS to mimic a variety of dermatoses. A high index of suspicion, supported by histopathology is required to diagnose and treat it without delay to avoid complications. We hope that it will help with the knowledge and better understanding of its atypical variants.

## CONFLICT OF INTEREST STATEMENT

None to declare.

## AUTHOR CONTRIBUTIONS

**Mario Magaña**: Resources (equal); supervision (equal); visualization (equal); writing – review & editing (equal). **Carol Estefania Márquez‐Maldonado**: Conceptualization (equal); investigation (equal); writing – original draft (equal); writing – review & editing (equal).

## ETHICS STATEMENT

Written informed consent was obtained from the individual for the publication of any potentially identifiable images or data included in this article. It includes use in social media.

## Data Availability

The data underlying this article will be shared on reasonable request to the corresponding author.
